# Translational Control of the SigR-Directed Oxidative Stress Response in *Streptomyces* via IF3-Mediated Repression of a Noncanonical GTC Start Codon

**DOI:** 10.1128/mBio.00815-17

**Published:** 2017-06-13

**Authors:** Morgan A. Feeney, Govind Chandra, Kim C. Findlay, Mark S. B. Paget, Mark J. Buttner

**Affiliations:** a Department of Molecular Microbiology, John Innes Centre, Norwich Research Park, Norwich, United Kingdom; b Department of Cell and Developmental Biology, John Innes Centre, Norwich Research Park, Norwich, United Kingdom; c School of Life Sciences, University of Sussex, Brighton, United Kingdom; Massachusetts Institute of Technology

**Keywords:** IF3, noncanonical start codon, oxidative stress, sigma factors, translation initiation

## Abstract

The major oxidative stress response in *Streptomyces* is controlled by the sigma factor SigR and its cognate antisigma factor RsrA, and SigR activity is tightly controlled through multiple mechanisms at both the transcriptional and posttranslational levels. Here we show that *sigR* has a highly unusual GTC start codon and that this leads to another level of SigR regulation, in which SigR translation is repressed by translation initiation factor 3 (IF3). Changing the GTC to a canonical start codon causes SigR to be overproduced relative to RsrA, resulting in unregulated and constitutive expression of the SigR regulon. Similarly, introducing IF3* mutations that impair its ability to repress SigR translation has the same effect. Thus, the noncanonical GTC *sigR* start codon and its repression by IF3 are critical for the correct and proper functioning of the oxidative stress regulatory system. *sigR* and *rsrA* are cotranscribed and translationally coupled, and it had therefore been assumed that SigR and RsrA are produced in stoichiometric amounts. Here we show that RsrA can be transcribed and translated independently of SigR, present evidence that RsrA is normally produced in excess of SigR, and describe the factors that determine SigR-RsrA stoichiometry.

## IMPORTANCE

In all sigma factor-antisigma factor regulatory switches, the relative abundance of the two proteins is critical to the proper functioning of the system. Many sigma-antisigma operons are cotranscribed and translationally coupled, leading to a generic assumption that the sigma and antisigma factors are produced in a fixed 1:1 ratio. In the case of *sigR*-*rsrA*, we show instead that the antisigma factor is produced in excess over the sigma factor, providing a buffer to prevent spurious release of sigma activity. This excess arises in part because *sigR* has an extremely rare noncanonical GTC start codon, and as a result, SigR translation initiation is repressed by IF3. This finding highlights the potential significance of noncanonical start codons, very few of which have been characterized experimentally. It also emphasizes the limitations of predicting start codons using bioinformatic approaches, which rely heavily on the assumption that ATG, GTG, and TTG are the only permissible start codons.

## INTRODUCTION

Oxidative stress damages key macromolecules, including DNA and proteins. Therefore, the ability to respond to oxidative stress is critical for survival, and organisms have evolved diverse strategies to sense oxidative stress. Many of these rely on the reactivity of cysteine moieties in sensor proteins, with the oxidation or reduction of the cysteines having profound consequences for function. The activities of a number of transcription factors are controlled by thiol redox switches and regulate genes involved in oxidative stress responses (reviewed in references 1 and 2). For example, in the canonical redox-sensing transcription factor OxyR, formation of a reversible disulfide bridge between two conserved cysteines leads to large structural changes in the protein, and the oxidized form of OxyR activates a regulon of antioxidant genes (3, 4).

The principal oxidative stress response in *Streptomyces* is controlled by a regulatory system consisting of a sigma factor, SigR, and a redox-sensitive, SigR-specific antisigma factor, RsrA (5, 6). The antisigma factor contains one Zn(II) ion per monomer, coordinated by three cysteine residues and one histidine. Under reducing conditions, RsrA binds to SigR with nanomolar affinity and prevents it from associating with RNA polymerase to activate transcription (6, 7). When the cell encounters oxidative stress, an intramolecular disulfide bond forms in RsrA between two of the cysteines that coordinate the zinc ion, leading to expulsion of zinc and inactivation of RsrA as an antisigma factor (7–10). SigR is then released and activates the transcription of a regulon of more than 100 target genes (11–13).

The *trxBA* operon encoding a thioredoxin system and the genes specifying the biosynthesis of the thiol redox buffer mycothiol are key components of the SigR regulon (5, 14). Since the thioredoxin system and mycothiol are both able to reduce the intramolecular disulfide bond in oxidized RsrA, upregulation of these pathways restores the reducing environment of the cytosol and leads to reduction of RsrA to its active state (6, 14). RsrA then rebinds SigR, thereby shutting off SigR-dependent transcription and completing a homeostatic feedback loop.

When streptomycetes grow in the absence of oxidative stress, the *sigR-rsrA* operon is expressed from the promoter *sigRp1*. However, when they are exposed to oxidative stress and SigR is released from RsrA, it activates transcription of the *sigR-rsrA* operon from an upstream autoregulatory promoter, *sigRp2* (5). From the *p2* transcript, a longer isoform of the protein (SigR′) is translated from an upstream ATG start codon (15). Unlike the SigR isoform, which is stable, this SigR′ isoform is unstable because the 58-residue N-terminal extension present in SigR′ (but absent from SigR) renders it a substrate for Clp proteases, also members of the SigR regulon. This provides a second negative feedback loop, allowing the cell to dampen the SigR response once the stress has been dealt with and homeostasis has been restored (15).

In addition to the autoregulatory feedback loop described above, the expression of SigR is regulated at the transcriptional level in response to a number of antibiotics that specifically target the ribosome and interfere with protein synthesis (16). In response to antibiotic stress, the transcriptional regulator WblC induces the expression of the *sigRp1* transcript and the synthesis of the shorter, stable isoform of the protein, SigR. Thus, SigR expression and activity are tightly controlled at a number of levels.

In addition to the components of the thioredoxin and mycoredoxin/mycothiol pathways, the SigR regulon also includes other proteins involved in the reduction of oxidized proteins such as methionine sulfoxide reductases, proteins involved in protein quality control such as chaperones and proteases, and others involved in transcription and translation (11–13). The latter class includes translation initiation factor 2 (IF2) and IF3, encoded by the *infB* and *infC* genes, respectively (13).

Translation IF3 is an essential protein in bacteria that functions as an initiation fidelity factor, repressing translation from noncanonical start codons by destabilizing 30S initiation complexes that include start codons other than ATG, GTG, and TTG (17). The gene encoding IF3 itself (*infC*) is a rare example of a well-characterized gene with a noncanonical start codon (18–23). *infC* has an ATT start codon in *Escherichia coli* (ATC in *Streptomyces*), and thus, IF3 autoregulates its own translation. When IF3 levels fall, translation of IF3 increases, leading to higher levels of IF3 and rerepression, thereby forming a translational homeostatic feedback loop (18, 21).

Here we show that *sigR* has a highly unusual GTC start codon and that this leads to another level of SigR regulation, in which SigR translation is repressed by IF3. Changing the GTC to a canonical start codon or introducing mutations that impair IF3 functioning causes SigR to be overproduced relative to its antisigma factor RsrA, resulting in unregulated and constitutive expression of the SigR regulon. We also show that *rsrA* has its own dedicated promoter and that RsrA is produced through a combination of incomplete translational coupling to *sigR* and *sigR*-independent translation arising from ribosome entry at a dedicated *rsrA* ribosome binding site (RBS). The net result of these arrangements is that RsrA is produced in excess over SigR.

## RESULTS

### *sigR* uses a noncanonical GTC start codon.

**FIG 1:**
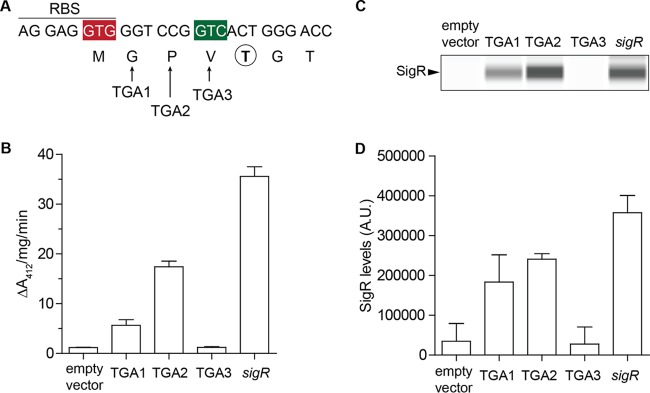
*sigR* uses a noncanonical GTC start codon. (A) Sequence showing the RBS, the annotated (red) and actual (green) start codons of *sigR*, the positions of the three introduced stop codons, and the experimentally determined first amino acid (circled and bold) of mature SigR purified from natural abundance from *S. coelicolor* (5). (B) Disulfide reductase activities in Δ(*sigR-rsrA*) null strain J2146 with the empty vector pSET152 and in J2146 with pSET152 carrying the wild-type *sigR* gene or parallel constructs with the annotated second (GGT), third (CCG), or fourth (GTC) *sigR* codon changed to TGA (stop). In the absence of RsrA, SigR drives high levels of expression of multiple thioredoxin genes, and the resulting disulfide reductase activity was measured by monitoring the reduction of the synthetic, disulfide-containing chromogenic substrate DTNB, which leads to the appearance of TNB (412 nm). (C) Automated Western blot analysis showing the effect of codon changes on SigR levels, generated with the quantitative Wes capillary electrophoresis and blotting system (ProteinSimple, San Jose, CA; see Materials and Methods). The same amount (2.5 µg) of total protein of each sample was loaded, and SigR was detected with an anti-SigR polyclonal antibody. A single replicate of each strain is shown. (D) Quantitation of SigR levels (area under each peak; arbitrary units [A.U.]). All samples were analyzed in triplicate, and the mean value and standard error of each sample are shown.

The annotated start codon of *Streptomyces coelicolor sigR* is GTG (Fig. 1A). Two facts made us doubt this assignment. First, this GTG codon is inappropriately positioned because it is internal to the extensive predicted AGGAGGTG *sigR* RBS (Fig. 1A). Second, when we purified SigR from natural abundance in *S. coelicolor* and subjected it to Edman degradation, the N-terminal sequence was TGTDAGTEH (in four separate preparations [5]). If the GTG codon were correctly assigned as the start codon, this would imply that four N-terminal residues (fMGPV) were removed posttranslationally, which seemed unlikely. To address this issue, we individually changed the annotated second, third, and fourth codons (GGT CCG GTC) to a TGA stop codon, introduced the resulting *sigR* alleles into a Δ(*sigR-rsrA*) null strain (J2146), and assayed disulfide reductase activity as a proxy for SigR activity [in the absence of RsrA, SigR drives high levels of expression of multiple thioredoxin genes, and the resulting disulfide reductase activity can be monitored by measuring the reduction of the synthetic, disulfide-containing, chromogenic substrate 5,5′-dithiobis(2-nitrobenzoic acid) (DTNB)]. SigR was still active when the GGT or CCG codon was mutated, but changing the GTC codon abolished SigR activity *in vivo* (Fig. 1B). We also analyzed these strains by Western blotting and found that changing the GTC codon to TGA abolished SigR production, whereas SigR was still produced when the GGT or CCG codon was changed to TGA (Fig. 1C and D). From these results, we concluded that SigR has a highly unusual GTC start codon and that the N-terminal fMet that it encodes is removed posttranslationally to leave mature SigR with a TGTDAGTEH N terminus (Fig. 1A).

### The noncanonical GTC *sigR* start codon is critical for correct and proper functioning of the oxidative stress regulatory system.

**FIG 2:**
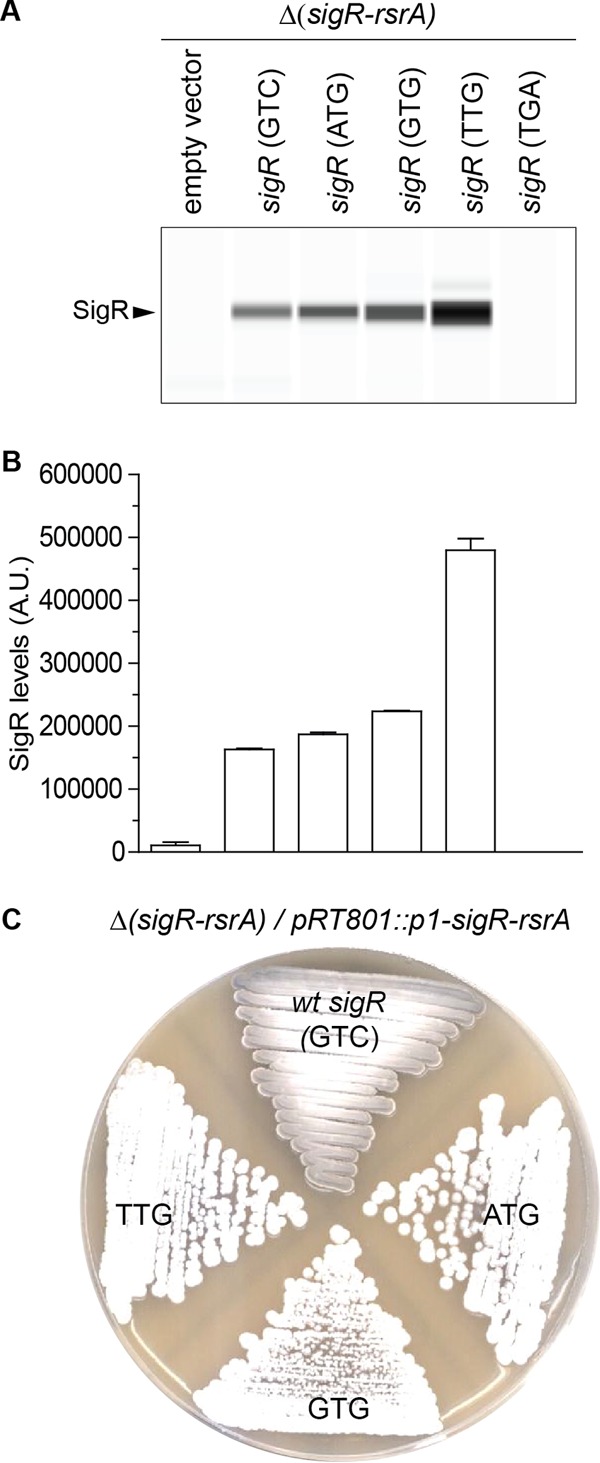
The noncanonical GTC *sigR* start codon is critical for correct and proper functioning of the oxidative stress regulatory system. (A) Automated Wes Western blot showing SigR levels in Δ(*sigR-rsrA*) null strain J2146 carrying the empty pRT801 integrative vector or parallel pRT801 constructs carrying the *p1-sigR-rsrA* operon with a GTC (wild type), ATG, GTG, or TTG start codon. The same amount (2.5 µg) of total protein of each sample was loaded, and SigR was detected with an anti-SigR polyclonal antibody. A single replicate of each strain is shown. (B) Quantitation of SigR levels (area under each peak; arbitrary units [A.U.]). All samples were analyzed in triplicate, and the mean value and standard error of each sample are shown. (C) Changing the natural *sigR* GTC start codon to any of the canonical start codons (ATG, GTG, or TTG) blocks sporulation, resulting in white colonies because of the absence of the gray spore pigment. Shown are colonies of Δ(*sigR-rsrA*) null strain J2146 carrying the empty pRT801 integrative vector or parallel pRT801 constructs carrying the *p1-sigR-rsrA* operon with a GTC (wild type), ATG, GTG, or TTG start codon grown on SFM and photographed after 4 days. *wt*, wild type.

To determine the biological importance of the GTC start codon of *sigR*, we changed it to canonical start codons. To focus solely on SigR, we used an integrative plasmid that carried the *sigR-rsrA* operon expressed from just the *p1* promoter (*p1-sigR-rsrA*). This prevented expression of the longer SigR′ isoform, which is only expressed from the upstream *p2* autoregulatory promoter. We introduced parallel constructs differing only in the *sigR* start codon into the *S. coelicolor* Δ(*sigR-rsrA*) null strain (J2146). Initially, we tested the effects on these mutations on SigR expression by Western blotting and found that when GTC was changed to any of the canonical start codons (ATG, GTG, or TTG), SigR was overexpressed relative to the wild-type GTC start codon (Fig. 2A and B). Unexpectedly, we consistently saw that a TTG start codon led to higher levels of SigR than either ATG or GTG. This echoes the findings of Myronovskyi et al. (24), who found that TTG also serves as a more efficient start codon than ATG or GTG for the *gus* reporter gene.

Regulation of the SigR-RsrA system is of critical importance to the biology of the organism, as shown by the fact that uncontrolled SigR activity (SigR > RsrA) blocks *Streptomyces* sporulation, causing the colonies to display a characteristic “white” (Spo^−^) phenotype due to the lack of synthesis of the gray spore pigment. Although the reasons for this phenotype are not understood (one possibility is competition between SigR and sporulation-specific sigma factors for core RNA polymerase), it provides an extremely convenient visual means to assay SigR activity (25). Δ*sigR* Δ*rsrA* double mutants sporulate normally (Spo^+^), showing that the developmental defect of the Δ*rsrA* single mutant is due to unchecked SigR activity (25). We therefore also looked at the effects of the start codon changes on sporulation and found that whereas the control construct carrying *p1-sigR-rsrA* with the wild-type GTC *sigR* start codon did not alter the gray (Spo^+^) phenotype of J2146, changes in canonical start codons each caused a full white (Spo^−^) phenotype (Fig. 2C). We concluded that changing the *sigR* GTC start codon to a canonical start codon causes SigR to be produced in excess over RsrA (SigR > RsrA). Thus, the *sigR* GTC start codon is a critical component of the oxidative stress response, and the SigR-RsrA regulatory system fails to function properly without it.

### SigR and RsrA are not produced at equimolar levels, and *rsrA* can be translated independently of *sigR*.

**FIG 3:**
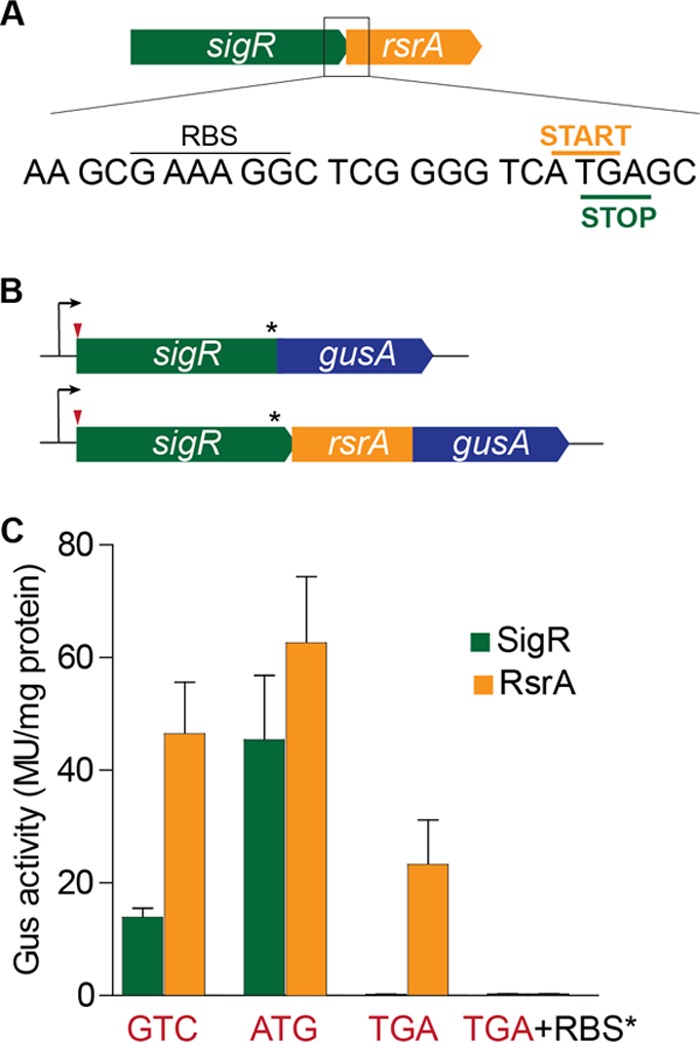
SigR and RsrA are not produced at equimolar levels, and *rsrA* can be translated independently of *sigR*. (A) Sequence showing the *rsrA* RBS and the overlap between the *sigR* stop codon and the *rsrA* start codon. (B) The* sigR-gusA* and *rsrA-gusA* translational reporter fusions used. The positions of the mutations made in the *sigR* start codon and the *rsrA* RBS are indicated by the red arrowhead and the asterisk, respectively. (C) Relative SigR and RsrA expression levels when the *sigR* start codon is GTC (wild type), ATG, or TGA (stop) and when the *rsrA* RBS is mutated in combination with a TGA* sigR* start codon. The *rsrA* RBS mutation used was GAAAGG to TCTAGA. MU, Miller units.

As is often the case in sigma-antisigma operons (26, 27), the start codon of the downstream *rsrA* gene overlaps the stop codon of the upstream *sigR* gene in the ATGA manner (Fig. 3A). As a consequence, it has been assumed that the two genes are translationally coupled (28, 29) to ensure that the two proteins are made in equimolar amounts and that all translation of *rsrA* would depend on the prior translation of *sigR* (2, 6, 25, 30). The fact that changing the *sigR* start codon to ATG caused a white (Spo^−^) phenotype, as described above, implied that the translational coupling of *sigR* and *rsrA* is incomplete, such that the increase in SigR translation was not reflected in an equal increase in the level of RsrA translation (SigR > RsrA).

To address this question experimentally, we used a version of the *gusA* (β-glucuronidase) reporter gene codon optimized for use in *Streptomyces* (24). These experiments were performed with *Streptomyces venezuelae* because *S. coelicolor* produces the intensely blue-pigmented antibiotic actinorhodin, which interferes with the detection of Gus activity with the chromogenic substrate 5-bromo-4-chloro-3-indolyl-β-d-glucuronic acid (X-Gluc). We made a full-length translational fusion of *sigR* to *gusA* (to exclude any autoregulatory effects, we did not include the SigR-dependent *sigRp2* promoter, so that the fusion protein was transcribed only from the *sigRp1* promoter) (Fig. 3B). We introduced this *sigR-gusA* translational reporter and an equivalent *rsrA-gusA* translational fusion (Fig. 3B) into wild-type *S. venezuelae* and found higher levels of expression with the *rsrA-gus* fusion construct than with the *sigR-gus* construct (Fig. 3C). Furthermore, we found that mutation of the SigR GTC start codon to ATG caused an ~4-fold increase in *sigR-gus* expression but had little effect on the expression of the equivalent *rsrA-gus* construct (Fig. 3C), again suggesting that translational coupling of *sigR* and *rsrA* was incomplete. Through mutagenesis of the *sigR* start codon to a TGA stop codon, we showed that all *sigR* translation depended on the presence of its GTC start codon, as expected. However, we unexpectedly found that ~50% of *rsrA* translation still occurred in the absence of *sigR* translation (Fig. 3C).

Seeking to account for this observation, we identified an appropriately spaced putative RBS (GAAAGG) 9 bp upstream of the *rsrA* ATG start codon (Fig. 3A). Mutation of the putative *rsrA* RBS in the construct carrying the GTC-to-TGA change at the *sigR* start codon abolished all remaining *rsrA* translation (Fig. 3C). Thus, RsrA appears to be produced through a combination of incomplete translational coupling to *sigR* and *sigR*-independent translation arising from ribosome entry at a dedicated *rsrA* RBS. Taken together, these results suggested that, in the absence of oxidative stress, RsrA is produced in excess over SigR and SigR is sequestered as a result.

To investigate this issue further, we introduced the ATG*sigR-rsrA* operon into the wild-type strain (M600). Whereas we had previously found that this allele generated a white (Spo^−^) phenotype in a Δ(*sigR-rsrA*) null strain (J2146), we found that the white phenotype of the ATG*sigR-rsrA* allele was recessive to the wild-type chromosomal GTC*sigR-rsrA* locus (see Fig. S1 in the supplemental material). This finding implies that RsrA is normally produced in excess of SigR and that sufficient free RsrA is produced from the native GTC*sigR-rsrA* locus to sequester the excess SigR derived from the ATG*sigR-rsrA* allele in the merodiploid strain.

10.1128/mBio.00815-17.1 FIG S1  The ATG*sigR-rsrA* allele is recessive to the wild-type GTC*sigR-rsrA* allele. Shown are wild-type *S. coelicolor* and congenic Δ(*sigR-rsrA*) null strain J2146 with the pRT801 integrative vector carrying the *p1-sigR-rsrA* operon with either the wild-type *sigR* GTC start codon or an engineered ATG start codon. Strains were grown on SFM and photographed after 4 days. Download FIG S1, EPS file, 0.5 MB. Copyright © 2017 Feeney et al. 2017 Feeney et al. This content is distributed under the terms of the Creative Commons Attribution 4.0 International license.

**FIG 4:**
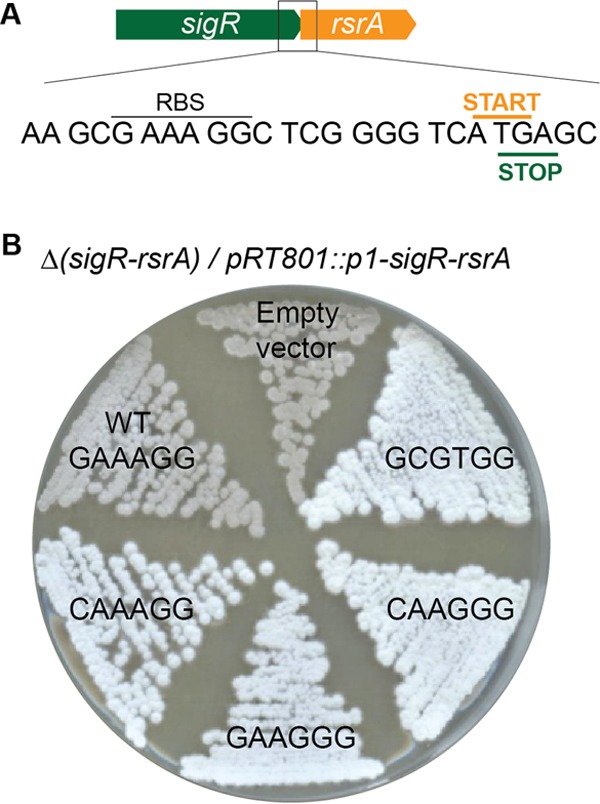
Weakening of the *rsrA* RBS results in overexpression of SigR relative to RsrA. (A) Sequence showing the *rsrA* RBS and the overlapping stop and start codons of *sigR* and *rsrA*, respectively. (B) Mutation of the *rsrA* RBS blocks sporulation, resulting in white colonies because of the absence of the gray spore pigment. Shown are colonies of Δ(*sigR-rsrA*) null strain J2146 with the empty pRT801 integrative vector or with parallel pRT801 constructs carrying the *p1-sigR-rsrA* operon with the wild-type (WT) *rsrA* RBS or the mutant RBS sequences indicated. One of these RBS mutations (GAAAGG to GCGTGG) resulted in a conservative arginine-to-lysine substitution in SigR, while the other three did not alter the amino acid sequence of SigR. Strains were grown on SFM and photographed after 4 days.

To test whether the *rsrA* RBS and *sigR*-independent translation of RsrA are important for the production of RsrA in excess over SigR, we next made four separate mutations to weaken the RBS (Fig. 4). Three of these RBS mutations did not alter the amino acid sequence of SigR, while the fourth (GAAAGG to GCGTGG) resulted in a conservative arginine-to-lysine substitution in SigR. We introduced these mutations into the *p1-sigR-rsrA* operon carried on integrative plasmid pRT801 and moved the resulting constructs into the Δ(*sigR-rsrA*) null strain (J2146). In each case, the mutant RBS allele conferred a white (Spo^−^) phenotype, suggesting that SigR was being produced in excess over RsrA, whereas the Δ(*sigR-rsrA*) null strain carrying the wild-type *p1-sigR-rsrA* allele sporulated normally, as expected (Fig. 4B).

### *rsrA* can be transcribed independently of *sigR.*

**FIG 5:**
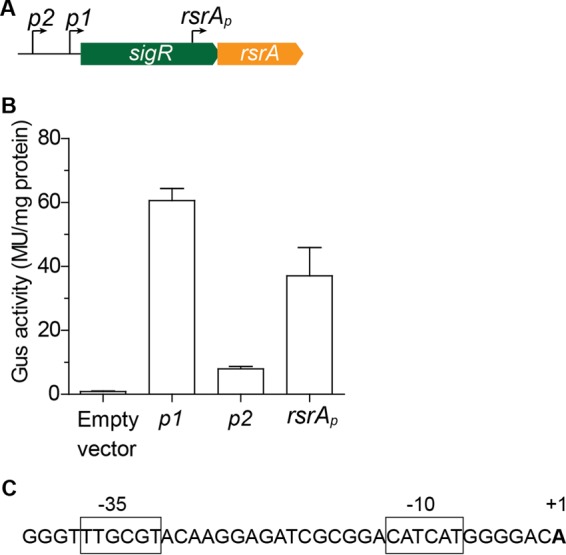
*rsrA* has its own dedicated promoter internal to the *sigR* coding sequence. (A) Schematic showing the relative positions of the *sigRp2* (autoregulatory), *sigRp1*, and *rsrAp* promoters. (B) Gus activity driven by each of the three promoters individually (these are basal levels; the *sigRp2* autoregulatory promoter can be induced by oxidative stress). These assays were conducted with *S. venezuelae* because *S. coelicolor* produces the blue-pigmented antibiotic actinorhodin, which interferes with the detection of Gus activity with the chromogenic substrate X-Gluc. (C) Sequence of the newly identified *rsrAp* promoter, with the transcription start site and putative −35 and −10 sequences highlighted. MU, Miller units.

The *sigR-rsrA* operon has long been known to be transcribed from the *sigRp1* and *sigRp2* promoters upstream of the *sigR* gene (Fig. 5A). Realizing that *rsrA* had its own RBS, we then wondered whether it might also be transcribed independently of *sigR* from a dedicated promoter internal to the *sigR* gene. We therefore cloned the 300 bp upstream of the *rsrA* start codon into a *gus* promoter probe vector, pIJ10742. As controls, we also cloned the *sigRp1* and *sigRp2* promoters individually into the same vector. We then tested the promoter activity of these three fragments in *S. venezuelae* and found that all three drove β-glucuronidase expression (Fig. 5B). Expression from the SigR-dependent *sigRp2* promoter was relatively low under these noninducing conditions. Expression from the *rsrAp* promoter was lower than that from the *sigRp1* promoter but higher than that from the uninduced *sigRp2* autoregulatory promoter.

We determined the precise location of the *rsrAp* promoter as part of a genome-wide transcription start site mapping experiment. The *rsrAp* transcription start site sits within the 300-bp fragment cloned into the *gus* promoter probe vector, lying downstream of −10 and −35 elements likely to be recognized by the principal, essential sigma factor in *Streptomyces*, HrdB (Fig. 5C).

### *sigR* translation is regulated by IF3.

**FIG 6:**
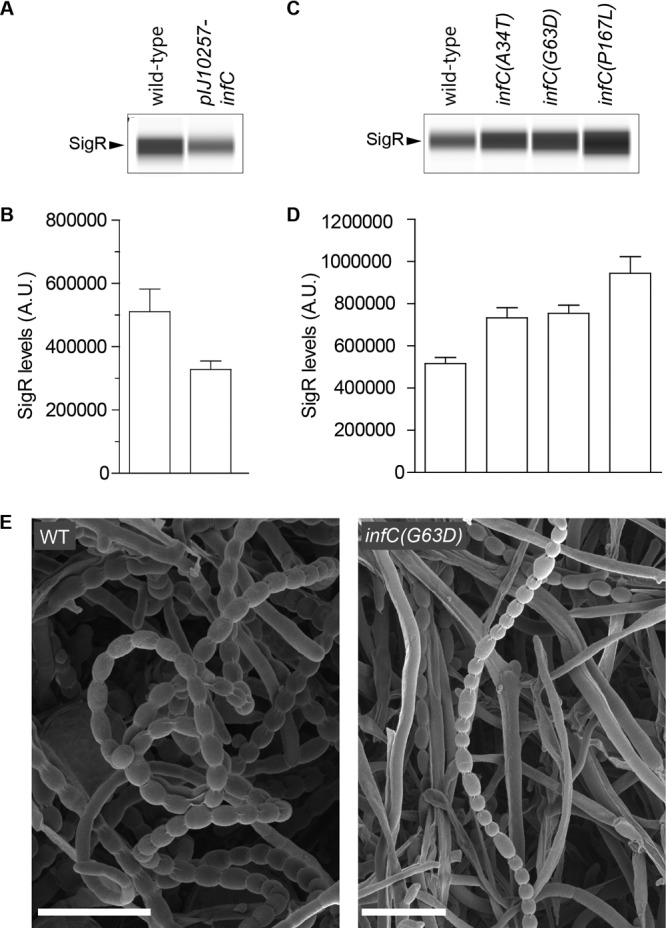
SigR translation is regulated by IF3. (A) Automated Wes Western blot showing that a second copy of *infC* suppresses SigR levels. (B) Quantitation of SigR levels in panel A (area under each peak; arbitrary units [A.U.]). (C) Automated Wes Western blot showing that the introduction of an infC* fidelity mutation (A34T, G63D, or P167L) into the native *infC* locus increases SigR levels. (D) Quantitation of SigR levels in panel C (area under each peak; arbitrary units [A.U.]). For panels A to D, all samples were analyzed in triplicate and the mean value and standard error of each sample are shown. The same amount (2.5 µg) of total protein of each sample was loaded, and SigR was detected with an anti-SigR polyclonal antibody. In the Western blot assays, a single replicate of each strain is shown. (E) Scanning electron micrographs of wild-type (WT) *S. coelicolor* and a congenic *infC* (G63D) mutant. Scale bars, 5 μm. Strains were grown on SFM and imaged after 6 days.

A number of *in vitro* and *in vivo* studies have shown that IF3 represses translation from noncanonical start codons in *E. coli* (18–23, 31, 32). To determine if IF3 also represses translation from the GTC start codon of *sigR* in *S. coelicolor*, we increased IF3 levels by introducing a second copy of *infC*, expressed from a strong constitutive promoter (ermE*p) on an integrative plasmid. This overexpression of IF3 caused a decrease in SigR levels, consistent with the idea that IF3 represses SigR translation (Fig. 6A and B).

Although *infC* is an essential gene, a variety of viable E. coli infC* fidelity mutants with impaired IF3 functioning have been isolated that, depending on their severity, result in a 3- to 15-fold increase in translation from noncanonical start codons (18). Since several of the IF3 residues affected in these E. coli infC* strains are conserved in *Streptomyces* IF3 (see Fig. S2), we reconstructed three of these mutations as *S. coelicolor* alleles and introduced them at the native *infC* locus, creating *S. coelicolor* strains that produced only an IF3* mutant protein. Western blotting showed that SigR was expressed at higher levels in each of the three infC* fidelity mutant strains than in the wild type (Fig. 6C and D). The infC* fidelity mutants showed a partial white (Spo^−^) phenotype, producing a mixture of undifferentiated hyphae with occasional irregular spore chains (Fig. 6E).

10.1128/mBio.00815-17.2 FIG S2  infC* mutant alleles constructed in *S. coelicolor*. Shown is a pairwise alignment of the *S. coelicolor* and *E. coli* IF3 homologs with the mutated residues highlighted in red. The *S. coelicolor* mutant alleles constructed were A34T, G63D, and P167L, corresponding to A42T, G71D, and P176L in *E. coli* IF3. Download FIG S2, EPS file, 0.3 MB. Copyright © 2017 Feeney et al. 2017 Feeney et al. This content is distributed under the terms of the Creative Commons Attribution 4.0 International license.

### The regulatory elements of the SigR-RsrA system are highly conserved across *Streptomyces* species.

We looked across 34 sequenced *Streptomyces* genomes to determine if the various regulatory elements described above are conserved (see Table S1). The extensive *sigR* RBS (AGGAGGTG) was highly conserved across the 34 *sigR* homologs. The *sigR* GTC start codon was present in 32 of the 34 *Streptomyces* species, but *Streptomyces griseoflavus* and *Streptomyces griseorubens* instead had a GTT codon at this position. It therefore seems likely that *sigR* has a noncanonical GTT start codon in a minority of *Streptomyces* species. The *rsrAp* promoter was readily identifiable in all 34 homologs. While the −10 sequence was invariant, a number of differences were found in the −35 sequence, the most prominent of which, TTGCG instead of TTGCC, accounted for nearly half of the sequences examined. Finally, the *S. coelicolor rsrA* RBS (GAAAGG) was the same in 33 species but was GGAAGG in *Streptomyces xiamenensis*.

10.1128/mBio.00815-17.4 TABLE S1  Conservation of regulatory elements in *sigR-rsrA*. Download TABLE S1, DOCX file, 0.02 MB. Copyright © 2017 Feeney et al. 2017 Feeney et al. This content is distributed under the terms of the Creative Commons Attribution 4.0 International license.

## DISCUSSION

**FIG 7:**
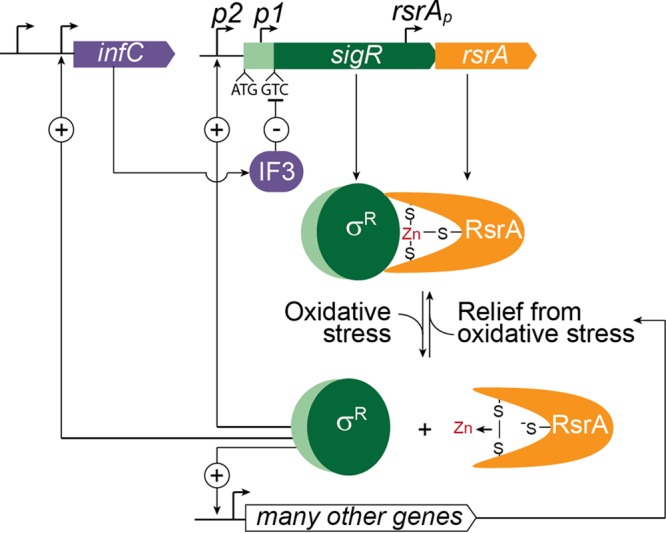
Regulation of the oxidative stress response in *Streptomyces*. The oxidative stress response is controlled by sigma factor SigR and zinc-containing, redox-sensitive antisigma factor RsrA. Under reducing conditions, RsrA binds SigR and prevents it from activating transcription. Under these conditions, the *sigR-rsrA* operon is only expressed from the *p1* promoter and only the short isoform of SigR (dark green) is synthesized. The *sigR* gene encoding this short isoform has a highly unusual GTC start codon, and this leads to repression of SigR translation by IF3. This translational repression is essential to prevent SigR from being overproduced relative to RsrA, which would result in unregulated and constitutive expression of the SigR regulon. An excess of RsrA over SigR is ensured through a combination of (i) incomplete translational coupling to *sigR*, and (ii) independent transcription and translation of *rsrA* arising from its own dedicated promoter and RBS, both internal to the *sigR* coding sequence. Exposure to oxidative stress induces the formation of an intramolecular disulfide bond in RsrA and the expulsion of zinc, which causes it to lose its affinity for SigR, releasing SigR to activate the transcription of >100 genes and operons, including the IF3 structural gene* infC* (which has additional promoters that do not depend on SigR). SigR also activates the transcription of the *sigR-rsrA* operon from upstream autoregulatory promoter* p2*. Translation of the *p2* transcript leads to the synthesis of a longer isoform of the protein (SigR′) from an upstream ATG start codon lying between the two promoters. Unlike the stable SigR isoform, SigR′ is unstable because the N-terminal extension found only in SigR′ (pale green) makes it a substrate for Clp proteases, which are also members of the SigR regulon. This provides a second negative feedback loop controlling SigR activity.

In all sigma-antisigma regulatory switches, the relative abundance of the two proteins is critical to the proper functioning of the system. We describe here a novel mechanism of translational control of the sigma factor SigR and its cognate antisigma factor RsrA that may have broader implications for understanding the expression of other sigma-antisigma pairs. We show that *sigR* has a highly unusual GTC start codon and that this leads to repression of SigR translation by IF3 (Fig. 7). Replacement of the GTC codon with any of the three canonical start codons (ATG, GTG, TTG) causes SigR to be produced in excess of RsrA, resulting in constitutive, uncontrolled expression of the SigR regulon. In the same way, IF3* mutations that impair the ability of IF3 to repress noncanonical start codons also result in an excess of SigR over RsrA and unchecked expression of the oxidative stress response. Thus, the noncanonical GTC *sigR* start codon and its repression by IF3 are essential for correct and proper functioning of the oxidative stress regulatory switch in *Streptomyces*. To our knowledge, *sigR* is the first documented example of a gene that uses a GTC start codon.

IF3 functions to repress translation initiation at noncanonical start codons. It binds to the small (30S) ribosomal subunit and regulates the fidelity with which the mRNA start codon and initiator tRNA substrates are selected into the 30S initiation complex. IF3 exists in a dynamic equilibrium between different conformational states, and recognition of a proper anticodon-codon interaction between the fMet initiator tRNA and the start codon in the 30S initiation complex shifts this equilibrium toward a single conformation of IF3. This conformation promotes progress along the initiation pathway and stabilizes contacts with the 50S subunit that lead to the formation of the 70S initiation complex and the elongation phase of protein synthesis (32, 33). It seems possible that the very extensive RBS (AGGAGGTG) upstream of the *sigR* GTC start codon helps to stabilize the otherwise unfavorable formation of an initiation complex containing the fMet initiator tRNA mispaired with the GTC codon. However, it is clear that the kinetics of translation initiation at the *sigR* start codon and the role of IF3, particularly under oxidative stress conditions, merit further study.

Previously, it had been assumed that all transcription of *rsrA* arose from the *p1* and *p2* promoters that lie upstream of *sigR*, that *sigR* and *rsrA* were translationally coupled via the ATGA arrangement of their respective stop and start codons, and that all translation of *rsrA* would depend on translation of the upstream *sigR* gene, leading to production of the two proteins in a fixed 1:1 stoichiometry. Here we have shown that RsrA is produced in excess over SigR and that this excess is ensured through a combination of (i) incomplete translational coupling to *sigR* and (ii) independent transcription and translation of *rsrA* arising from its own dedicated promoter and RBS, both internal to the *sigR* coding sequence. The *rsrA* promoter and RBS sequences are highly conserved across *Streptomyces* species, and mutation of the *rsrA* RBS results in SigR expression in excess over RsrA and constitutive expression of the SigR regulon.

The SigR-RsrA system was first characterized in *Streptomyces*, but it is present throughout the actinomycetes, including important clinical pathogens such as *Mycobacterium tuberculosis* and *Corynebacterium diphtheriae*. The SigR-RsrA (SigH-RshA) system has been shown to be important for the pathogenesis of *M. tuberculosis* (34), presumably because it helps pathogens to resist oxidative killing by white blood cells. While the noncanonical GTC start codon is well conserved across *Streptomyces sigR* homologs, there is no evidence of its use in these other actinobacterial genera. Both the *M. tuberculosis* and *C. glutamicum sigH* genes are annotated as having canonical ATG start codons, and in the case of *M. tuberculosis*, a large-scale proteomic analysis identified a peptide corresponding to this annotated start site (35). In *C. glutamicum*, the ATG codon is downstream of an appropriately placed RBS, supporting its annotation as the start codon.

*infC*, the gene encoding IF3, has several promoters, one of which is a direct target of SigR (13) (Fig. 7). This raised the possibility that a previously unrecognized translational homeostatic feedback loop could regulate SigR activity, in which IF3 functioning was directly or indirectly compromised by oxidative stress, leading to derepression of the *sigR* GTC start codon, a transient excess of SigR over RsrA, and increased expression of the SigR regulon, including the gene encoding IF3. This speculative possibility seemed especially attractive given that Cys65 of *E. coli* IF3 is known to become oxidized *in vivo* during hypochlorite stress (36), suggesting that IF3 might sense oxidative stress directly. Further, this cysteine is conserved in *Streptomyces* IF3. However, to date, we have not been able to demonstrate enhanced translation of SigR from the GTC start codon in response to oxidative stress. It will be important in the future, therefore, to examine the translation of SigR under a broader range of different oxidative conditions and to understand the biological rationale behind the wiring of a regulatory network that places SigR directly under the control of IF3.

Our data suggest that SigR translation from its noncanonical GTC start codon is limited by IF3 and that independent transcription and translation of *rsrA* ensure that RsrA is present in excess of SigR, providing a buffer to prevent spurious release of SigR activity. In this context, it is interesting to note that the antisigma factor has also been shown to be more abundant than the sigma factor in two other systems, *E. coli* SigE/RseA and FliA/FlgM (37). Further, translation of *E. coli sigE* has been shown to be limited by the second codon, and the kinetics of the stress response can be altered by changes in the basal level of SigE expression (38). Many sigma-antisigma factor pairs are encoded by cotranscribed and overlapping open reading frames, which are assumed to be translationally coupled. To query whether the translational imbalance seen in the SigR-RsrA switch might be a more general phenomenon in sigma-antisigma systems, we examined the sequences of 16 other putative sigma-antisigma operons from the *S. coelicolor* genome. In 15 of 16 cases, we found a candidate RBS appropriately positioned upstream of the start codon of the downstream putative antisigma factor gene (see Fig. S3), suggesting that the antisigma factor can be translated independently of the sigma factor in each of these cases.

10.1128/mBio.00815-17.3 FIG S3  Potential antisigma RBSs in 17 putative sigma-antisigma operons taken from the *S. coelicolor* genome. The sigma factor stop codon is red, the start codon of the putative antisigma factor is in bold, and putative antisigma RBSs are underlined. Download FIG S3, EPS file, 0.5 MB. Copyright © 2017 Feeney et al. 2017 Feeney et al. This content is distributed under the terms of the Creative Commons Attribution 4.0 International license.

Finally, our work highlights the limitations of predicting start codons using bioinformatic approaches, which rely heavily on the assumption that ATG, GTG, and TTG are the only permissible start codons. As an illustration, the *sigR* start codon is misannotated to a nearby upstream canonical start codon in all available streptomycete genomes, and the *infC* start codon is similarly misannotated in most *Streptomyces* species, even though the use of a noncanonical start codon by *infC* is well documented in the literature. It seems likely that the experimental identification of start codons through global ribosome profiling will be the most effective way to circumvent this difficulty (37, 39) and thus generate an accurate picture of the totality of genes subject to IF3-mediated translational repression.

## MATERIALS AND METHODS

### Bacterial strains, plasmids, oligonucleotides, and culture conditions.

The strains, plasmids, and oligonucleotides used in this study are listed in Table S2. *S. coelicolor* strains (M600 and derivatives) were routinely grown on SFM agar or in TSB/YEME liquid medium (40); *S. venezuelae* was routinely grown on MYM agar or in liquid MYM medium (41). Where indicated, oxidants were added to the growth medium to induce oxidative stress. Conjugation was used to introduce cosmids and plasmids into *Streptomyces* as described previously (40).

10.1128/mBio.00815-17.5 TABLE S2  Strains, plasmids, and oligonucleotides used in this study. Download TABLE S2, DOCX file, 0.02 MB. Copyright © 2017 Feeney et al. 2017 Feeney et al. This content is distributed under the terms of the Creative Commons Attribution 4.0 International license.

### Introduction of unmarked point mutations into the chromosomal *infC* locus.

To construct an infC* mutant strain, we developed a simple method for introducing unmarked point mutations into the chromosome of *Streptomyces*. First, we used PCR targeting (42, 43) to introduce an *apr-oriT* cassette (pIJ794) into a cosmid (StI35) containing our gene of interest (*infC* in this case), replacing the kanamycin resistance marker already present on the cosmid backbone. Second, we replaced the wild-type *infC* allele with the mutant infC* alleles. To do this, we took advantage of the fact that *E. coli* strains deficient in mismatch repair (*mutS*) have a very high frequency of lambda red-mediated recombination of single-stranded DNA, high enough that there is no need for a selectable marker (44). We transformed the *mutS* recombineering strain HME68 carrying the *apr-oriT* derivative of cosmid StI35 with a 60-mer oligonucleotide carrying the desired point mutation. We then screened for candidates with the directed point mutation either by PCR (based on primers binding to the mutant allele but not the wild type) or PCR followed by diagnostic restriction digestion.

Once we had identified cosmids carrying the desired mutation, we transformed DH5α with them to generate a clonal population, isolated mutant cosmid DNA, and confirmed the presence of the mutation (and the lack of extraneous mutations) by sequencing of *infC*. The mutant cosmids were then moved into the recipient *Streptomyces* strain, selecting first for introduction of the cosmid (apramycin resistance). After restreaking several times under nonselective conditions, we screened for apramycin-sensitive derivatives in which a double crossover had occurred. We observed that roughly half of the double-crossover exconjugants had a developmental defect. Using the same approach described above (mismatch PCR or PCR of *infC* followed by diagnostic restriction digestion), we confirmed that the white exconjugants were infC* mutants while the gray (Spo^+^) exconjugants were *infC*^+^ (wild type). We also sequenced the *infC* allele in three or four exconjugants to confirm the results of the diagnostic PCRs.

### Cloning and site-directed mutagenesis.

Standard techniques were used for PCR amplification, restriction digestion, and cloning, and QuikChange mutagenesis was used to introduce point mutations in accordance with the Stratagene protocol. All constructs were sequenced (MWG Eurofins).

### *gusA* reporter constructs.

To create a pMS82-*gusA* reporter plasmid, the fragment containing *aadA*, its flanking transcription terminator upstream of the *gusA* gene multiple cloning site, and *gusA* was excised from the pGUS plasmid (24) as a PvuII fragment and then subcloned into the EcoRV site of pMS82 (45). Next, site-directed mutagenesis was used to introduce unique NdeI and XbaI sites into the *gusA* multiple cloning site, generating pIJ10742. This vector was used as a negative control in *gus* assays. We also generated a positive control, where the *gus* gene was under the control the of ermE*p promoter, as follows. First, the ermE*p promoter was excised from pIJ10257 (46) with Asp718 and XbaI, gel purified, and ligated into the pGUS vector digested with Asp718 and SpeI. The *gus* reporter, including the upstream *aadA* gene flanked by transcriptional terminators, was then excised with PvuII and subcloned into similarly digested pMS82 (45), generating pIJ10741 (pMS82-ermE*p-*gus*). To construct transcriptional fusions, the *sigRp1* (pIJ10850), *sigRp2* (pIJ10851), and *rsrAp* (pIJ10881) promoter fragments were amplified from *S. venezuelae* genomic DNA by PCR with the oligonucleotides listed in Table S2, digested with NdeI and XbaI, and ligated into similarly digested pIJ10742. In all of these constructs, the *gus* reporter gene is expressed with its own RBS and ATG start site.

The translational GusA reporters were subcloned from pGUS-HL4aadA (23) into pMS82 (45) by the following approach. First, *p1*-*sigR* and *p1*-*sigR-rsrA* were amplified by PCR from *S. venezuelae* 5KO8 cosmid DNA with primers Sven-SigR-Gus1/Sven-SigR-Gus2 and Sven-SigR-Gus1/Sven-RsrA-Gus2. The PCR products were digested with XbaI and EcoRV and then ligated into similarly digested pGUS-HL4aadA, generating constructs where the *gusA* gene was fused to the last codon of the *sigR* and the *rsrA* genes, respectively. QuikChange mutagenesis was then used to mutate the *sigR* start codon to either ATG or TGA in both the *sigR-gus* and *rsrA-gus* constructs. These constructs were then subcloned into pMS82 by PCR amplification of the reporter construct (including the upstream flanking *aadA* gene and transcriptional terminator preventing readthrough into the *gus* gene) with the oligonucleotides Gus1-NsiI and Gus2-KpnI, digestion with NsiI and KpnI, and ligation into similarly digested pMS82.

### *sigR*-*rsrA* constructs.

For the experiments shown in Fig. 1, inverse PCR was used to introduce TGA mutations into *sigR* in pIJ5981 (25), which contains *sigR* together with both upstream promoters but lacks the downstream *rsrA* gene. The *sigR* mutant genes were then cloned as XbaI fragments into pSET152 and introduced into *S. coelicolor* J2146 (Δ*sigR*-*rsrA*) by conjugation.

For subsequent experiments, vectors for expression of the *sigR*-*rsrA* operon were constructed as follows. First, the entire operon (including the *sigRp1* promoter but excluding the autoregulatory *sigRp2* promoter) was amplified by PCR from St7E4 cosmid DNA with primers Sco-p1SigR-BamHI and ScoRsrArev-EcoRV, digested with BamHI and EcoRV, and ligated into similarly cut pRT801. This generated pIJ10863, where the *sigR-rsrA* operon is expressed from its own *sigRp1* promoter and *sigR* is translated from its native GTC start codon. QuikChange mutagenesis was then performed to change this GTC start codon to either a canonical start codon or a stop codon: Sco-SigR-ATG (pIJ10864), Sco-SigR-TTG (pIJ10865), Sco-SigR-GTG (pIJ10866), and Sco-SigR-TGA (pIJ10867).

### Wes analysis.

For analysis of SigR protein levels, we used the quantitative Wes capillary electrophoresis and blotting system (ProteinSimple, San Jose, CA) with the Wes-Rabbit (12 to 230 kDa) Master kit and Split buffer (Biotechne). Wes uses an automated, capillary-based method for immunodetection of proteins. Protein samples were prepared for Wes in accordance with the manufacturer’s directions. We determined the optimal conditions for α-SigR Wes and showed, by using purified protein, that the signal was within the linear range. Cell lysates were diluted to a final protein concentration of 0.5 mg/ml, and the α-SigR antibody was used at a dilution of 1:400. For each experiment, samples were assayed in triplicate (technical replicates). Data were analyzed with the Compass software (version 2.6.7).

### Disulfide reductase assays.

*S. coelicolor* strains were grown in NMMP (40) supplemented with 1% (wt/vol) glucose to an optical density at 450 nm of 1.0 (mid to late log phase). The mycelium was harvested by centrifugation, washed in ice-cold 0.9% NaCl, resuspended in ice-cold lysis buffer (50 mM Tris-HCl [pH 7.5] 150 mM NaCl, 1 mM EDTA), disrupted by sonication, and then centrifuged at 16,000 × *g* for 10 min to obtain a cell-free lysate. Disulfide reductase assays were performed with DTNB as described previously (5).

### β-Glucuronidase activity assays.

For qualitative analysis of Gus reporter fusions, *S. venezuelae* strains expressing the relevant constructs were grown on MYM agar containing trace elements and 0.16 mg/ml X-Gluc (Gold Biotechnologies). Where necessary, oxidants were added to paper disks and placed on the growth medium to induce oxidative stress in the reporter strains. To quantify the expression of reporter constructs, β-glucuronidase assays were performed as described previously (24), done in triplicate, and repeated at least three times.

### Scanning electron microscopy.

Scanning electron microscopy was performed as described previously (47).
