# Supplementary material for: Translational Control of the SigR-Directed Oxidative Stress Response in Streptomyces via IF3-Mediated Repression of a Noncanonical GTC Start Codon
Source: mBio. 2017 Jun 13;8(3):e00815-17. doi: 10.1128/mBio.00815-17 (PMC5472188; doi:10.1128/mBio.00815-17)
Supplement: TABLE S1 [file mbo003173348st1.docx]

**Table S1. Conservation of regulatory elements in *sigR-rsrA***

| Organism | Genome | SigR hit | SigR RBS | SigR start | RsrA promoter | RsrA RBS |
| --- | --- | --- | --- | --- | --- | --- |
| Streptomyces coelicolor A3(2) | NC_003888 | Score = 1270 bits (1408), Expect = 0.0  Identities = 704/704 (100%), Gaps = 0/704 (0%) | AGGAGGTG | GTC | -10 TTGCC  -35 CATCAT | GAAAGG |
| Streptomyces albulus strain NK660 | NZ_CP007574 | Score = 841 bits (932), Expect = 0.0  Identities = 625/733 (85%), Gaps = 46/733 (6%) | x+ | + | TTGCG  + | + |
| Streptomyces albus strain DSM 41398 | NZ_CP010519 | Score = 836 bits (926), Expect = 0.0  Identities = 606/700 (87%), Gaps = 6/700 (1%) | + | + | TTGCG  + | + |
| Streptomyces albus J1074 | NC_020990 | Score = 825 bits (914), Expect = 0.0  Identities = 607/711 (85%), Gaps = 33/711 (5%) | + | + | +  + | + |
| Streptomyces avermitilis MA-4680 | NC_003155 | Score = 1076 bits (1192), Expect = 0.0  Identities = 659/701 (94%), Gaps = 0/701 (0%) | + | + | TTGCG  + | + |
| Streptomyces bingchenggensis BCW-1 | NC_016582 | Score = 843 bits (934), Expect = 0.0  Identities = 618/715 (86%), Gaps = 20/715 (3%) | + | + | +  + | + |
| Streptomyces cattleya DSM 46488 | NC_017586 | Score = 821 bits (910), Expect = 0.0  Identities = 609/711 (86%), Gaps = 24/711 (3%) | + | + | TTCGC  + | + |
| Streptomyces clavuligerus ATCC 27064 | NZ_CM000913 | Score = 818 bits (906), Expect = 0.0  Identities = 599/703 (85%), Gaps = 30/703 (4%) | + | + | +  + | + |
| Streptomyces collinus Tu 365 | NC_021985 | Score = 1056 bits (1170), Expect = 0.0  Identities = 654/700 (93%), Gaps = 0/700 (0%) | + | + | TTGCG  + | + |
| Streptomyces davawensis strain JCM 4913 | NC_020504 | Score = 1072 bits (1188), Expect = 0.0  Identities = 661/706 (94%), Gaps = 6/706 (1%) | + | + | TTGCG  + | + |
| Streptomyces glaucescens strain GLA.O | NZ_CP009438 | Score = 1041 bits (1154), Expect = 0.0  Identities = 659/715 (92%), Gaps = 15/715 (2%) | + | + | TTGCG  + | + |
| Streptomyces griseoflavus Tu4000 | NZ_GG657758 | Score = 1070 bits (1186), Expect = 0.0  Identities = 660/704 (94%), Gaps = 3/704 (0%) | + | GTT | TTGCG  + | + |
| Streptomyces griseorubens strain JSD-1 | NZ_KL503830 | Score = 1036 bits (1148), Expect = 0.0  Identities = 652/703 (93%), Gaps = 3/703 (0%) | + | GTT | +  + | + |
| Streptomyces hygroscopicus ATCC 53653 | NZ_GG657754 | Score = 810 bits (898), Expect = 0.0  Identities = 614/719 (85%), Gaps = 25/719 (3%) | + | + | TTGCG  + | + |
| Streptomyces hygroscopicus subsp. jinggangensis | NC_017765 | Score = 1052 bits (1166), Expect = 0.0  Identities = 660/712 (93%), Gaps = 12/712 (2%) | + | + | +  + | + |
| Streptomyces iranensis | NZ_LK022848 | Score = 877 bits (972), Expect = 0.0  Identities = 625/715 (87%), Gaps = 22/715 (3%) | + | + | +  + | + |
| Streptomyces leeuwenhoekii | NZ_LN831790 | Score = 1133 bits (1256), Expect = 0.0  Identities = 672/701 (96%), Gaps = 0/701 (0%) | + | + | +  + | + |
| Streptomyces lydicus A02 | NZ_CP007699 | Score = 787 bits (872), Expect = 0.0  Identities = 569/654 (87%), Gaps = 9/654 (1%) | + | + | TTCCG  + | + |
| Streptomyces niveus NCIMB 11891 | NZ_CM002280 | Score = 850 bits (942), Expect = 0.0  Identities = 607/704 (86%), Gaps = 30/704 (4%) | + | + | +  + | + |
| Streptomyces nodosus strain ATCC 14899 | NZ_CP009313 | Score = 1067 bits (1182), Expect = 0.0  Identities = 657/701 (94%), Gaps = 0/701 (0%) | + | + | +  + | + |
| Streptomyces pratensis ATCC 33331 | NC_016114 | Score = 865 bits (958), Expect = 0.0  Identities = 608/701 (87%), Gaps = 27/701 (4%) | + | + | +  + | + |
| Streptomyces pristinaespiralis strain HCCB 10218 | NZ_CP011340 | Score = 866 bits (960), Expect = 0.0  Identities = 610/701 (87%), Gaps = 18/701 (3%) | + | + | TTGCG  + | + |
| Streptomyces purpureus KA281 | NZ_KB913030 | Score = 866 bits (960), Expect = 0.0  Identities = 611/704 (87%), Gaps = 33/704 (5%) | + | + | TTGCG  + | + |
| Streptomyces roseochromogenes subsp. oscitans DS  12.976 | NZ_CM002285 | Score = 1047 bits (1160), Expect = 0.0  Identities = 658/712 (92%), Gaps = 12/712 (2%) | + | + | TTGCG  + | + |
| Streptomyces scabiei 87.22 | NC_013929 | Score = 996 bits (1104), Expect = 0.0  Identities = 640/701 (91%), Gaps = 12/701 (2%) | + | + | TTGCG  + | + |
| Streptomyces sp. 150FB | NZ_JTHL01000001 | Score = 778 bits (862), Expect = 0.0  Identities = 592/704 (84%), Gaps = 30/704 (4%) | + | + | +  + | + |
| Streptomyces sp. CdTB01 | NZ_CP013743 | Score = 1050 bits (1164), Expect = 0.0  Identities = 653/700 (93%), Gaps = 0/700 (0%) | + | + | TTGCG  + | + |
| Streptomyces sp. CNQ-509 | NZ_CP011492 | Score = 825 bits (914), Expect = 0.0  Identities = 615/716 (86%), Gaps = 18/716 (3%) | + | + | +  + | + |
| Streptomyces sp. Tu6071 | NZ_CM001165 | Score = 899 bits (996), Expect = 0.0  Identities = 623/703 (89%), Gaps = 21/703 (3%) | + | + | +  + | + |
| Streptomyces sviceus ATCC 29083 | NZ_CM000951 | Score = 1038 bits (1150), Expect = 0.0  Identities = 650/700 (93%), Gaps = 0/700 (0%) | + | + | TTGCG  + | + |
| Streptomyces venezuelae ATCC 10712 | NC_018750 | Score = 875 bits (970), Expect = 0.0  Identities = 613/704 (87%), Gaps = 33/704 (5%) | + | + | TTGCG  + | + |
| Streptomyces vietnamensis strain GIM4.0001 | NZ_CP010407 | Score = 893 bits (990), Expect = 0.0  Identities = 617/704 (88%), Gaps = 33/704 (5%) | + | + | TTGCG  + | + |
| Streptomyces violaceusniger Tu 4113 | NC_015957 | Score = 845 bits (936), Expect = 0.0  Identities = 618/715 (86%), Gaps = 22/715 (3%) | + | + | +  + | + |
| Streptomyces viridochromogenes DSM 40736 | NZ_GG657757 | Score = 1113 bits (1234), Expect = 0.0  Identities = 669/703 (95%), Gaps = 3/703 (0%) | + | + | +  + | + |
| Streptomyces xiamenensis strain 318 | NZ_CP009922 | Score = 760 bits (842), Expect = 0.0  Identities = 537/613 (88%), Gaps = 7/613 (1%) | + | + | +  + | GGAAGG |
