# Supplementary material for: Translational Control of the SigR-Directed Oxidative Stress Response in Streptomyces via IF3-Mediated Repression of a Noncanonical GTC Start Codon
Source: mBio. 2017 Jun 13;8(3):e00815-17. doi: 10.1128/mBio.00815-17 (PMC5472188; doi:10.1128/mBio.00815-17)
Supplement: TABLE S2 [file mbo003173348st2.docx]

**Table S2. Strains, plasmids and oligonucleotides used in this study**

| **Strain/plasmid** | **Relevent genotype/comments** | **Source/reference** |
| --- | --- | --- |
| ***S. coelicolor* A3(2)** |  |  |
| M600 | SCP1^-^ SCP2^-^ | Chakraburtty and Bibb (1997) |
| J2146 | M600 *Δ*(*sigR*-*rsrA*)::*hyg* (Hyg^R^) | Paget *et al*. (2001) |
| J3435 | M600 *infC*(A34T) | This work |
| J3436 | M600 *infC*(G63D) | This work |
| J3437 | M600 *infC*(P167L) | This work |
| J3438 | J2146 *infC*(A34T) | This work |
| J3439 | J2146 *infC*(P167L) | This work |
| ***S. venezuelae*** |  |  |
| ATCC10712 | wild-type |  |
| SV40 | Δ*sigR::apr* (Apr^R^) | This work |
| SV41 | Δ*rsrA::apr* (Apr^R^) | This work |
| SV42 | Δ*(sigR-rsrA)::apr* (Apr^R^) | This work |
| ***E. coli*** |  |  |
| ET12567(pUZ8002) |  | Paget *et al*. (1999) |
| HME68 | §W3110 *galK*tyr145UAG Δ*lac*U169 [λ cI857 Δ(*cro-bioA*)] *mutS*<>cat | Costantino and Court (2003) |
| TB10 | *rph-1 ilvG rfb-50* λΔ*cro-bio nad*::Tn*10* | Johnson *et al*. (2004) |
|  |  |  |
| **Plasmids** |  |  |
| pSET152 | ΦC31 conjugative integrative vector (Apr^R^) | Bierman *et al*. (1992) |
| pIJ773 | apramycin cassette for gene replacement | Gust *et al*. (2004) |
| pMS82 | ΦBT1 conjugative integrative vector (Hyg^R^) | Gregory *et al.* (2003) |
| pRT801 | ΦBT1 conjugative integrative vector (Apr^R^) | Gregory *et al.* (2003) |
| pGus | *gus* reporter vector for transcriptional fusions | Myronovskyi *et al.* (2011) |
| pGusHL4aadA | *gus* reporter vector for translational fusions |  |
| pIJ10257 | ΦBT1 conjugative integrative vector (Hyg^R^) containing the *ermE**p promoter | Hong *et al.* (2005) |
| pIJ12551 | ΦC31 conjugative integrative vector (Apra^R^) containing the *ermE**p promoter | Sherwood *et al*. (2013) |
|  | ***gusA* translational fusions** |  |
| pIJ10743 | p1*sigR*-*gusA* (wild-type GTC start codon) (ΦBT1, Hyg^R^) | This work |
| pIJ10744 | p1*sigR*-*gusA* (ATG start codon) (ΦBT1, Hyg^R^) | This work |
| pIJ10745 | p1*sigR*-*gusA* (TAG start codon) (ΦBT1, Hyg^R^) | This work |
| pIJ10746 | p1*sigR…rsrA-gusA* (*sigR* GTC start codon) (ΦBT1, Hyg^R^) | This work |
| pIJ10747 | p1*sigR…rsrA-gusA* (*sigR* ATG start codon) (ΦBT1, Hyg^R^) | This work |
| pIJ10748 | p1*sigR…rsrA-gusA* (*sigR* TAG start codon) (ΦBT1, Hyg^R^) | This work |
| pIJ10880 | p1*sigR…rsrA-gusA* (*sigR* TAG start codon, *rsrA* RBS^-^) (ΦBT1, Hyg^R^) | This work |
|  | ***gusA* transcriptional fusions** |  |
| pIJ10881 | *rsrA*p-*gusA* (ΦBT1, Hyg^R^) | This work |
| pIJ10850 | *sigR*p1-*gusA* (ΦBT1, Hyg^R^) | This work |
| pIJ10851 | *sigR*p2-*gusA* (ΦBT1, Hyg^R^) | This work |
| pIJ10741 | pMS82-*ermE**p-*gusA* (ATG start codon) (ΦBT1, Hyg^R^) | This work |
| pIJ10742 | pMS82-*gusA* (ΦBT1, Hyg^R^) | This work |
|  | ***sigR* vectors** |  |
| pIJ10863 | pRT801-p1*sigRrsrA* (*sigR* start codon the wild-type GTC) (ΦBT1, Apr^R^) | This work |
| pIJ10864 | pRT801-p1*sigRrsrA* (ATG *sigR* start codon) (ΦBT1, Apr^R^) | This work |
| pIJ10865 | pRT801-p1*sigRrsrA* (TTG *sigR* start codon) (ΦBT1, Apr^R^) | This work |
| pIJ10866 | pRT801-p1*sigRrsrA* (GTG *sigR* start codon) (ΦBT1, Apr^R^) | This work |
| pIJ10867 | pRT801-p1*sigRrsrA* (TGA *sigR* start codon) (ΦBT1, Apr^R^) | This work |
|  |  |  |
|  | ***infC* vectors** |  |
| pIJ10882 | pIJ10257-*infB* (ΦBT1, Hyg^R^) | This work |
| pIJ10883 | pIJ10257-*infC* (ΦBT1, Hyg^R^) | This work |
| pIJ10884 | pIJ12551-*rsrA*(ΦC31, Apra^R^) | This work |

**Oligonucleotides**

Restriction sites are indicated by underlined text; mutations introduced by QuikChange mutagenesis by capital letters.

| **Primer name** | **Primer sequence** |
| --- | --- |
| Sco-p1SigR-BamHI | atgcggatcccgccgacctggactggaccg |
| Sco-RsrArev-EcoRV | atgcgatatctcaggactcctgcggggccg |
| Sco-SigR-ATG-1 | tcggaggaggtgggtccgATGactgggaccgacgcaggg |
| Sco-SigR-ATG-2 | ccctgcgtcggtcccagtCATcggacccacctcctccga |
| Sco-SigR-TTG1 | tcggaggaggtgggtccgTTGactgggaccgacgcaggg |
| Sco-SigR-TTG2 | ccctgcgtcggtcccagtCAAcggacccacctcctccga |
| Sco-SigR-GTG1 | tcggaggaggtgggtccgGTGactgggaccgacgcaggg |
| Sco-SigR-GTG2 | ccctgcgtcggtcccagtCACcggacccacctcctccga |
| Sven-SigR-Gus1 | atcgtctagatccccagggtgcccgtattc |
| Sven-SigR-Gus2 | tgacgatatctgagcccgagcctttcagatc |
| Sven-RsrA-Gus2 | tgacgatatcctcctggggcgccggtgtcg |
| Sven-SigR-ATG1 | ctggaggaggtgggtccgATGactgggaccgacacaggg |
| Sven-SigR-ATG2 | ccctgtgtcggtcccagtCATcggacccacctcctccag |
| Sven-SigR-TGA1 | ctggaggaggtgggtccgTGAactgggaccgacacaggg |
| Sven-SigR-TGA2 | ccctgtgtcggtcccagtTCAcggacccacctcctccag |
| Sven-RsrA-RBS1 | agagtcgtcgaacgatctTCATCTctcgggctcatgagctgc |
| Sven-RsrA-RBS2 | gcagctcatgagcccgagAGATGAagatcgttcgacgactct |
| Gus1-NsiI | atgcatgcatagttaagccgcgccgcgaag |
| Gus2-KpnI | atgcggtacctcactgcttcccgccctgctg |
| gusMCS-Nde1 | aattctctagaggatccatATgtaccaagcttattggca |
| gusMCS-Nde2 | tgccaataagcttggtacATatggatcctctagagaatt |
| gusMCS-Xho1 | tgtaccaagcttattggcTcCagtcgagcaacggaggta |
| gusMCS-Xho2 | tacctccgttgctcgactGgAgccaataagcttggtaca |
| ScoInfC-A34T | gagcaggtcgggattgtcccgcttgccaagACGctggagcttgcgcaggagtacgacctcgac |
| ScoInfC-A34Trc | gtcgaggtcgtactcctgcgcaagctccagCgTcttggcaagcgggacaatcccgacctgctc |
| SvenSigRdel1 | gggtccgccctcgggctcgccgccgcgctggaggaggtgattccggggatccgtcgacc |
| SvenSigRdel2 | ccgagcaatccgtctcgtgcggctctccgcagctcatgatgtaggctggagctgcttc |
| SvenRsrAdel1 | agagtcgtcgaacgatctgaaaggctcgggctcatgagcattccggggatccgtcgacc |
| SvenRsrAdel2 | cgcgacggcctccgtctcaccccgcgtcggggggtgtcatgtaggctggagctgcttc |
| Sven-trxCp-NdeI | atgccatatgtcgttctcgtgcgcgtgcgc |
| Sven-trxCp-XhoI | atgcctcgaggcttcctacctttcggggtc |
| Sven-sigRp1-NdeI | atgccatatgtccccagggtgcccgtattc |
| Sven-sigRp1-XhoI | atgcctcgagatcgtctcgctccgctgacc |
| Sven-sigRp2-NdeI | atgccatatgatcgtctcgctccgctgacc |
| Sven-sigRp2-XhoI | atgcctcgagccagcgcggcggcgagcccg |
| Sven-rsrAp300-NdeI | atgccatatgtcgacggggctgcggtccgc |
| Sven-rsrAp-XhoI | atgcctcgagtgagcccgagcctttcagatc |
| Sco-sigRA219A-1 | cggagagtcgaacgaagcCaaaggctcggggtcatgagct |
| Sco-sigRA219A-2 | agctcatgaccccgagcctttGgcttcgttcgactctccg |
| Sco-sigRK220K-1 | cggagagtcgaacgaagcgaaGggctcggggtcatgagct |
| Sco-sigRK220K-2 | agctcatgaccccgagccCttcgcttcgttcgactctccg |
| Sco-sigR-A210AK220K-1 | cggagagtcgaacgaagcCaaGggctcggggtcatgagct |
| Sco-sigR-A210AK220K-2 | agctcatgaccccgagccCttGgcttcgttcgactctccg |
| Sco-sigRK220R-1 | cggagagtcgaacgaagcgCGTggctcggggtcatgagct |
| Sco-sigRK220R-2 | agctcatgaccccgagccACGcgcttcgttcgactctccg |
| Sco-infB1-NdeI | atgcatcatatggctaaggtccgggtctac |
| Sco-infB2-HindIII | atgcataagctttacacccgcggcttctcgcg |
| Sco-infC1-NdeI | atgcatcatatgagcgccgagccccgcatc |
| Sco-infC2-HindIII | atgcataagcttcacgcttcggcaggtgcctc |
| Sco-rsrA1-NdeI | atgaaccatatgagcgccggagagccgcac |
| Sco-rsrA2-EcoRV | atgagatatctcaggactcctgcggggccga |

**Supplemental references**

**Bierman M, Logan R, O'Brien K, Seno ET, Rao RN, Schoner BE.** 1992. Plasmid cloning vectors for the conjugal transfer of DNA from *Escherichia coli* to *Streptomyces* spp. Gene **116:** 43-9.

**Chakraburtty R, Bibb M.** 1997. The ppGpp synthetase gene (relA) of *Streptomyces coelicolor* A3(2) plays a conditional role in antibiotic production and morphological differentiation. J Bacteriol. **179:** 5854-61.

**Costantino N, Court** **DL.** 2003. Enhanced levels of λ Red-mediated recombinants in mismatch repair mutants. Proc Natl Acad Sci U S A **100:** 15748-15753.

**Gregory MA, Till R, Smith MCM.** 2003. Integration site for *Streptomyces* phage ΦBT1 and development of site-specific integrating vectors. J Bacteriol **185:** 5320-5323.

**Gust B, Chandra G, Jakimowicz D, Yuqing T, Bruton C, Chater KF.** 2004. Lambda red-mediated genetic manipulation of antibiotic-producing *Streptomyces*. Adv Appl Microbiol **54:** 107–128.

**Hong, HJ, Hutchings MI, Hill LM, Buttner MJ.** 2005. The role of the novel Fem protein VanK in vancomycin resistance in *Streptomyces coelicolor*. J Biol Chem **280:** 13055-13061.

**Johnson JE, Lackner LL, Hale CA, de Boer PA.** 2004. ZipA is required for targeting of ^D^MinC/DicB, but not ^D^MinC/MinD, complexes to septal ring assemblies in *Escherichia coli*. J Bacteriol **186:** 2418-29.

**Myronovskyi M, Welle E, Fedorenko V, Luzhetskyy A.** 2011. β-Glucuronidase as a sensitive and versatile reporter in actinomycetes. App Environ Microbiol **77:** 5370-5383.

**Paget MSB, Chamberlin L, Atrih A, Foster SJ, Buttner MJ**. 1999. Evidence that the extracytoplasmic function sigma factor, σ^E^, is required for normal cell wall structure in *Streptomyces coelicolor* A3(2). J. Bacteriol. **181:**204–211.

**Paget MSB, Bae J-B, Hahn M-Y, Li W, Kleanthous C, Roe J-H, Buttner MJ.** 2001. Mutational analysis of RsrA, a zinc-binding anti-sigma factor with a thiol–disulphide redox switch. Mol Microbiol **39:** 1036-1047.

**Sherwood EJ, Hesketh AR, Bibb MJ.** 2013. Cloning and analysis of the planosporicin lantibiotic biosynthetic gene cluster of *Planomonospora alba*. J Bacteriol **195:** 2309-21.
